# Bead-probe complex capture a couple of SINE and LINE family from genomes of two closely related species of East Asian cyprinid directly using magnetic separation

**DOI:** 10.1186/1471-2164-10-83

**Published:** 2009-02-19

**Authors:** Chaobo Tong, Baocheng Guo, Shunping He

**Affiliations:** 1 Laboratory of Fish Phylogenetics and Biogeography, Institute of Hydrobiology, Chinese Academy of Sciences, Wuhan 430072, PR China; 2 Graduate University of the Chinese Academy of Sciences, Beijing 100039, PR China

## Abstract

**Background:**

Short and long interspersed elements (SINEs and LINEs, respectively), two types of retroposons, are active in shaping the architecture of genomes and powerful tools for studies of phylogeny and population biology. Here we developed special protocol to apply biotin-streptavidin bead system into isolation of interspersed repeated sequences rapidly and efficiently, in which SINEs and LINEs were captured directly from digested genomic DNA by hybridization to bead-probe complex in solution instead of traditional strategy including genomic library construction and screening.

**Results:**

A new couple of SINEs and LINEs that shared an almost identical 3'tail was isolated and characterized in silver carp and bighead carp of two closely related species. These SINEs (34 members), designated HAmo SINE family, were little divergent in sequence and flanked by obvious TSD indicated that HAmo SINE was very young family. The copy numbers of this family was estimated to 2 × 10^5 ^and 1.7 × 10^5 ^per haploid genome by Real-Time qPCR, respectively. The LINEs, identified as the homologs of LINE2 in other fishes, had a conserved primary sequence and secondary structures of the 3'tail region that was almost identical to that of HAmo SINE. These evidences suggest that HAmo SINEs are active and amplified recently utilizing the enzymatic machinery for retroposition of HAmoL2 through the recognition of higher-order structures of the conserved 42-tail region. We analyzed the possible structures of HAmo SINE that lead to successful amplification in genome and then deduced that HAmo SINE, SmaI SINE and FokI SINE that were similar in sequence each other, were probably generated independently and created by LINE family within the same lineage of a LINE phylogeny in the genomes of different hosts.

**Conclusion:**

The presented results show the advantage of the novel method for retroposons isolation and a pair of young SINE family and its partner LINE family in two carp fishes, which strengthened the hypotheses containing the slippage model for initiation of reverse transcription, retropositional parasitism of SINEs on LINEs, the formation of the stem loop structure in 3'tail region of some SINEs and LINEs and the mechanism of template switching in generating new SINE family.

## Background

SINE and LINE are interspersed nucleotide repeats distributed widely in eukaryotic genomes and occupy a substantial fraction of genome. For example, Alu and LINE1 constitute more than 13% and 20% of human genome, respectively [1]. They proliferate and replicate themselves through a "copy and paste" mechanism called retroposition involving transcription of their genomic copies followed by reverse transcription of an RNA intermediate and resulting cDNAs reintegration at a new location into the genome host [2-5]. Therefore, SINE and LINE, together with processed pseudogenes, are classified as retrotransposons [6].

Many biologists are interested and make great effort to isolate and characterize many retroposons (LINE and SINE) in various organisms, because it is an indispensable step for addressing the question that how they originate and evolve as well as their functioning and impact on the evolution of eukaryotic genomes. So far, over 100 LINE and nearly 100 SINE families have been described to date in various eukaryotic genomes [7]. Moreover, retroposons insertions have proven to be nearly perfect tools for studies of phylogeny and population biology [8,9] and have been successfully used to resolve phylogenetic relationships among various groups of different taxonomic rank [10-15]. Especially so far SINEs appear to have gained novel functions, acting for example as enhancers or silencers that regulate the expression of preexisting functional genes [16-18].

Currently methods allowing for isolation of SINE and LINE from an unknown genome mostly depend on construction of genomic library and subsequently screening by colony hybridization method using probe specific to particular region of repeated elements. Here we propose and describe a new strategy and method used to isolate SINEs and LINEs rapidly, in which library construction and screening is completely eliminated. This method is based on hybridization capture of repetitive elements from digested genomic DNA in solution using biotinylated oligonucleotide probes which have been pre-attached to streptavidin magnetic beads. Subsequently, the captured probe-target DNA fragment complex immobilized on the magnetic beads were selected and separated from other non-complementary fragments by magnetic separation, then released and amplified by adapter polymerase chain reaction (PCR), finally the PCR products enriched for SINE and LINE were cloned directly into T-vector for sequencing. The whole procedure was completed within only about a week.

LINEs are approximately 4–7 kilobase pair (kbp) in length and encode an endonuclease (EN) and a reverse transcriptase (RT), both of which are required for LINE retrotransposition [19]. Luan *et al*.[20] proposed the "target-primed reverse transcription" (TPRT) as the mechanism of LINE retrotransposition, in which the LINE EN creates a nick in the DNA of the host genome and the RT synthesizes cDNA in situ using a 3' OH of the DNA generated by the nick as a primer. In contrast, SINEs are relatively short (about 100–500 bp) and non-autonomous retroposons without ORFs and so lack the machinery to replicate themselves. It is suggested that SINE has recruited the enzymatic machinery for retroposition from the corresponding LINE through the common "tail"sequence [19,20], based on the observation that the sequences of many couple of SINE and LINE pairs isolated from many organisms were similar in their 3'end regions [21-24]. This scenario is supported by recent experiments of retrotransposition assay of eel UnaL2 and human LINE1. UnaL2 can strictly recognize a specific sequence at their 3'tail and mobilize transcript that has the 3' tail of UnaSINE1 [25,26], whereas Human LINE L1 can mobilize human SINE Alu via the poly A tail (no such 3'end-specific region) at the 3'end [27,28].

The template switch during TPRT was proposed as possible mechanism to explain the formation of chimeric retrotranscripts from a full copy of U6 small nuclear RNA fused to the 3'terminus of L1 [29-31] and the observation that several SINE families have a common 5' half sequence but different 3'tails [32,33]. So maybe the process of how SINE acquired the tail of partner LINE also resulted from the template switch between LINE and other RNA of SINE-to-be during TPRT [21,29,34].

The two closely related species of East Asian cyprinids, silver carp (*Hypophthalmichthys molitrix*) and bighead carp (*Aristichthys nobilis*), are believed to have origined recently [35]. In the present paper, we successfully use magnetic-bead based system to isolate SINE and LINE in this two species by developing special protocol. The data show that the designated HAmoSINE family was successfully proliferated recently through borrowing the enzymatic machinery of partner LINEs for retrotransposition in the two genomes. After comparison and detail analysis, we deduced that the HAmo SINE, SmaI SINE and FokI SINE that are similar in sequence with each other, were probably generated independently through the switch template between the LINE2 and RNA of SINE-to-be in respective genome because of an existence of no-similar central region between them. At last, the advantages of this new method and possibility of combining with other protocols are provided.

## Methods

### DNA extraction

All species DNA was isolated from ethanol-fixed tissues (fins or muscle) by incubation with proteinase K followed by phenol/chloroform extraction [36].

### Preliminary PCR to detect and identify SINE

AB-PCR was performed in silver carp and bighead carp as described elsewhere [37]. The reaction mixture (100 μl) contained 10 ng of genomic DNA and two 12-nucleotide primers ("A": 5'-TRGCTCAGTGGT-3', "B":5'-GGRATYGAACYC-3') specific to A and B boxes of RNA pol III promoter consensus, respectively. After 27 PCR cycles (95°C, 1 min; 34°C, 1 min; 72°C, 30s), the amplified ~55 bp DNA fragments were isolated by electrophoresis in 5% agarose gel.

**Figure 1:**
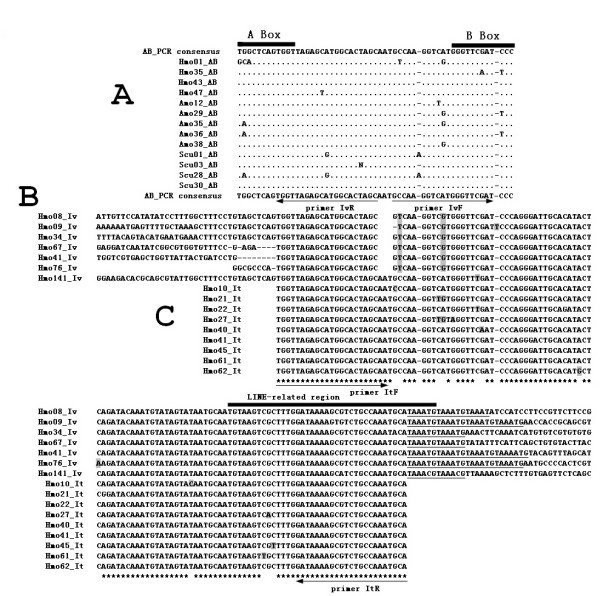
**The results of AB-PCR, Inverse PCR, internal-SINE PCR**. **Part A**: the aligned nucleotide sequences of the cloned AB-PCR products in silver carp and bighead carp. Hmo: *Hypophthalmichthys molitrix*, common name: silver carp; Amo: *Aristichthys mobilis*, common name: bighead carp; The corresponding consensus sequences are given below and above the sequences, respectively. The dots indicate nucleotides identical to the consensus sequences and the region of A box and B box are shown by thick bar. A pair of inverse primer (primer IvR and IvF) was designed to perform Inverse PCR. **Part B**: Inverse PCR sequences in silver carp that were obtained using primer IvR and IvF and recovered according to the procedure schemed and described in Additional file 1. The region of LINE2-related region followed by short tandem repeats are marked and underlined, respectively. Hmo141_Iv shows a completed SINE copies in amplified flanking region from a clone. A pair of internal primer (primer ItF and ItR) was designed to amplify many different SINE copies in silver carp. **Part C**: Alignment of internal region of many individual SINEs obtained using primer ItF and ItR (GenBank accession numbers: FJ171654-FJ171662). Nucleotides matched between three parts are denoted by asterisks. The different nucleotides are shaded.

Inverse PCR was carried out in silver carp as with modification of the method described elsewhere [38]. A pair of inverse primers: primer IF, primer IR (Figure 1, Additional file 1) was designed according to consensus sequence of AB-PCR fragments in silver carp and bighead carp. In brief, HaeIII-digested genomic DNA fragments were self-circularized in a final concentration of 5 ng/μl in a 100 μl ligation reaction. Inverse PCR (94°C, 1 min; 52°C, 1 min; 72°C, 2 min) was carried out using the 100 ng of above circularized DNA as template. The resulting smear fragments were cloned and sequenced.

A pair of primers (primer ItF, primer ItR, Figure 1) corresponding to internal region of SINE was used to detect many individual SINE copies. The PCR was run in a total volume of 20 μl including 200 ng DNA template with 25 cycles of 95°C 40s, 62°C 40s, 72°C 40s. Nine clones were selected randomly and sequenced. The clone Hmo41_It was used as probe to conduct the next retroposons enrichment strategy.

### Retroposons Enrichment Strategy

#### A. Preparation of genomic pool

##### 1) Digestion of genomic DNA

Approximately 40 mg of genomic DNA was completely digested with HaeIII (20 U/μl, Promega, Madison, WI, USA) overnight in a total volume of 100 μl. The fragmented DNA were subsequently separated by electrophoresis in 1% agarose. Fragments ranging from 700–2000 bp were purified from the gel using gel extraction kit (Omega) and finally suspended in 40 μl of H_2_O.

##### 2) Ligation of adapters

The adapter oligoA (5 P'-GGCAGGATCCACTGAATTCGC-3') and oligoB (5'-AGCGAATTCAGTGGATCCTGCC-3') were annealed by heating an equal volume of 10 μM oligonucleotides for 3 min at 95°C, 2 min at 65°C, 2 min at 45°C, 1 min at 25°C, conserved at 4°C. The annealed product is a double-stranded linker of which one end is blunt while the other has a 3' A overhang. Additionally, the oligoA was phosphorylated at the 5' base during manufacturing. Excess of annealed linkers were ligated to above 40 μl of prepared HaeIII-fragmented DNA (approximate 4 μg) in a 100 μl reaction containing 2 μM double-stranded linkers, l× ligase buffer, 20 units T4 DNA ligase (Fermentas, MBI), 10 μl 50% PEG4000. The reaction proceeded overnight at 22°C, and then purified through Takara column and resuspend in 150 μl H_2_O.

##### 3) PCR enrichment

Set up 20 PCR reactions with 28 μL ddH_2_O, 4.0 μL 10 mM dNTP's, 5 μL 10× PCR Buffer, 10 μL of the 2 μM oligo B primer and 0.5 μL of Taq each. Then add 1 μL of the linker ligation product to each PCR tube. The PCR reaction profile began with a 5 min 72°C filling in the nick between each linker and size fraction fragment left by the ligation step, then followed by 12 cycles of 95°C for 45s, 55°C for 45s, 72°C for 1 min 50 s. A 10 min extension step concluded the reaction. Then the total 20 tube PCR products were purified using 3–4 columns column in order not to overload the columns and finally resuspended in 150 μL H_2_O. The reason for doing the 16–24 separate PCR reaction at only 12 cycles is to maintain the complexity of the linker ligation mixture. Otherwise, a lot of identical clones would produce in the end. Before being used, the pool must be heat-denatured at 95°C for 10 min to make target single-strand DNA accessible to probe.

#### B. Preparation of bead-probe complex

##### 1) Probe biotinylation

Plasmid Hmo41_It corresponding to an internal region (18–144 bp) of an individual SINE was used as template to be biotinylated. In order to label one biotin at one terminus of the DNA fragment, we used the primers (primer ItF, ItR) with only primer ItF biotinylated to perform PCR. At last, the double strand PCR DNA with one strand biotinylated was purified in 100 μL H_2_O. Before bound to beads, the double strand biotinylated probe must be denatured at 95°C for 10 min.

##### 2) Probe bound to beads

Following the manufacturer's recommendation, 200 μL of Streptavidin Magnetic Particles (10 mg/ml, Roche, Mannheim, Germany) was collected by removing the storage buffer then washed three times with 300 μL binding buffer TEN100 (10 mM Tris-HCl, 1 mM EDTA, 100 mM NaCl, pH 7.5) for 5 min. Each time remove the supernatant using magnetic particle concentrator.

Above 100 μL denatured probe is added to beads of 300 μL binding buffer TEN100, then incubate for 30 min at room temperature to specifically bind the biotinylated strand, then removed supernatant containing non-biotinylated DNA strands followed by washing two times for 5 min with TEN100, then the preparation of the single strand probe-beads complex is accomplished.

#### C. Capture of target sequences

##### 1) Hybridization

The beads were washed once with 200 μL of hybridization buffer (5 × SSC, 0.1% SDS) for 5 min, then 150 μL of buffer (10 × SSC 0.2% SDS, preheated to 55°C) and 150 μL above denatured genomic pool were added to resuspend the beads followed by hybridization in 55°C for 2 hours, then non-complementary sequences were removed by washing successively with 400 μL TEN1000 (10 mM Tris-HCl, 1 mM EDTA, 1000 mM NaCl, pH 7.5) three times for 5 min; 400 μL buffer (0.2*SSC,0.1%SDS) three time for 5 min; 400 μL TEN1000 for 10 min, all these washing are conducted at room temperature, finally target DNA were release from the beads by elution at 95°C for 5 min in 50 μL H_2_O.

##### 2) Adapter PCR

Set up separate 4 PCR reactions with 14 μL ddH_2_O, 2.0 μL 10 mM dNTPs, 2.5 μL 10× PCR Buffer, 5 μL of the 2 μM Er1Bh1Blunt primer and 0.5 μL of Taq polymerase each. Then add 1 μL of the linker ligation product to each PCR tube. The PCR reaction profile began with a 5 min 94°C then followed by 15 cycles of 95°C for 45s, 55°C for 45s, 72°C for 1 min 50 s. A 10 min extension step concluded the reaction. Then the total 4 tube PCR products were purified for cloning.

##### 3) Cloning and Positive detection

The enriched PCR products were ligated directly into T- vector (Takara) using T4 DNA ligase, taking advantage of the 3'A overhangs often produced by Taq polymerase. Colony PCR amplification were performed directly on many single bacterial colony to determine the size of individual inserts, then select at random and sequence many clones in which inserted fragments were longer than 700 bp.

### Quantification of HAmo SINE copy number in two genomes using Quantitative Real Time-PCR

Plasmid Hmo41_It corresponding to one individual copy of HAmo SINE and Genomic DNA of silver carp and bighead carp were prepared as standard and sample for Real-Time PCR, respectively. Then, concentration of them were measured using spectrophotometer and five-fold serial dilutions of them were prepared respectively as templates to perform Real-Time PCR in a PCR machine (Bio-Rad, Chromo4) one time. All Real-Time PCR reactions was performed with 40 cycles at 95°C 40s, 62°C 40s, 72°C 40s including Primer ItF and ItR (300 nM final concentration) and SYBR GREEN in a final volume of 25 μL. At last, a melting curve analysis was done after the amplification phase. The standard curve and data analysis were carried out in the software MJ Opticon Monitor 3.1.

### Characterization of HAmo LINE family

To determine the 5' upstream sequence from a breakpoint at the HaeIII site of the HAmo LINE, we employed the method of genomic DNA walking in which TAIL-PCR (thermal asymmetric interlaced PCR) was conducted using one arbitrary degenerate prime provided by kit (Takara) and special primer designed according to the consensus of HaeIII-fragmented LINEs. The whole PCR processes are conducted according to manufacture's instruction and the last PCR products were cloned and sequenced.

## Results

### Preliminary PCR to detect and identify SINE

AB-PCR was amplified with very small amount of genomic DNA as a template and two oligonucleotides specific to boxes A and B of the promoter of RNA polymerase III as primers as described elsewhere [37]. Among many AB-PCR clones, we obtained some high similar sequences in two closely related species, silver carp and bighead carp, respectively (Figure 1A). The similar but not identical AB-PCR sequences and intact A box and B box, together with its reasonable sequence similarity to certain tRNAs (see below) indicated that they may have been amplified from different SINE copies of one same SINE family in the above two species.

To amplify the regions flanking AB-PCR fragment and test whether the above AB-PCR fragments belong to a part of a certain SINEs as we expected, a pair of primer: primer IvF and IvR which face in opposite orientations and correspond to consensus sequences of AB-PCR was designed to perform inverse PCR in silver carp (Figure 1A, Additional file 1). The whole procedure including recovery of origin sequence is shown schematically and described in Additional file 1. 7 of the recovered original sequences of inverse PCR shared a high similar region possessing characteristic features of typical SINEs, including LINE2-related region followed by short tandem repeats (TAAATG), but they differed in their flanking regions which indicate they may represent different retroposon locus (Figure 1B). These significant evidences implied that the shared region may be SINE and provided us a preliminary window to see the full structure of SINE.

In order to get the probe specific to the SINE for the next new non-library retroposons enrichment method, we designed a pair of primer (primer ItF, primer ItR) corresponding to the internal region of SINE to detect many individual SINE copies (Figure 1C). Nine clones selected by random show little sequence divergence indicate that this SINE family may be a young family. At last we selected plasmid Hmo41_It to be biotinylated as probe for the non-library enrichment strategy.

### Non-library Retroposons Enrichment Strategy

**Figure 2:**
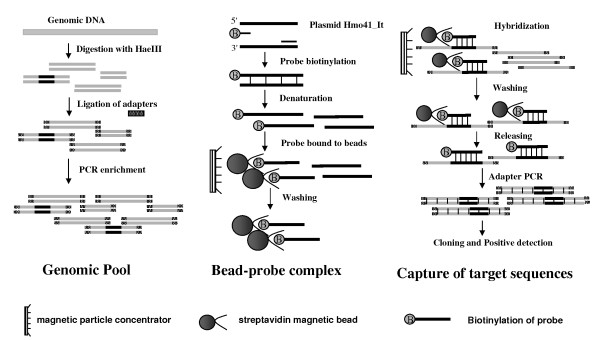
**Scheme of non-library retroposons enrichment strategy**. The whole protocol can be divided into three parts: preparation of genomic pool, preparation of bead-probe complex, capture of target sequences. Details in every step are described correspondingly in *Material and Methods *section.

**Figure 3:**
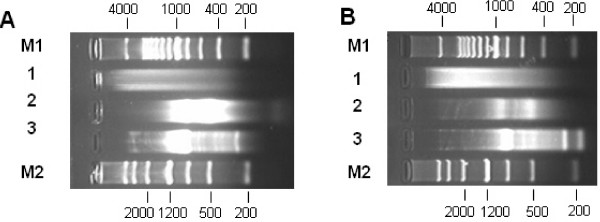
**Important phases monitored by Gel electrophoresis in the retroposons enrichment strategy in silver carp (A) and bighead carp (B)**. 1. Step *Digestion of genomic DNA*. HaeIII can cleave the genomic DNA and generate size fragments evenly (< 4 Kb). 2. Step *PCR enrichment*. 30 cycles of step PCR enrichment detected the size range of PCR-enriched HaeIII-fragmented DNA, and finally, after being optimized, only 12 cycles of the PCR enrichment was conducted for maintaining the complexity of DNA molecules. The enriched HaeIII-fragmented pool was manly ranging from 500–2000 bp. 3. Step *Adapter PCR*. 30 cycles of *Adapter PCR *detected the size of captured target fragments cloning into vector, and finally, after being optimized, only 15 cycles was done to keep the complexity of DNA molecules.

The Non-library Retroposons Enrichment Strategy has been conducted using plasmid Hmo41_It corresponding to the internal region of an individual SINE copy as probe to directly capture the HaeIII-fragmented genomic DNA containing the SINE sequence in solution. The whole procedure was schemed in Figure 2 and described in the *Materials and Methods *section. The entire isolation procedure can be completed in about a week. Every important phase can be monitored by the electrophoresis (Figure 3). At last, captured specific DNA fragments were cloned and sequenced and most of them were 700–1100 bp in length (Figure 3). In this case, the efficiency of the non-library strategy reaches nearly 60% by calculating the ratio of unique positive clones (Additional file 2). Finally, 51 and 29 SINE loci are determined in silver carp and bighead carp, respectively. Simultaneously, 28 LINE elements (HaeIII-fragmented LINEs and 5' truncated LINEs) were isolated in the final products, because the probe contained an about 40 bp tail region shared by SINE and LINE.

### Identification of young SINE family in silver carp and bighead carp

**Figure 4:**
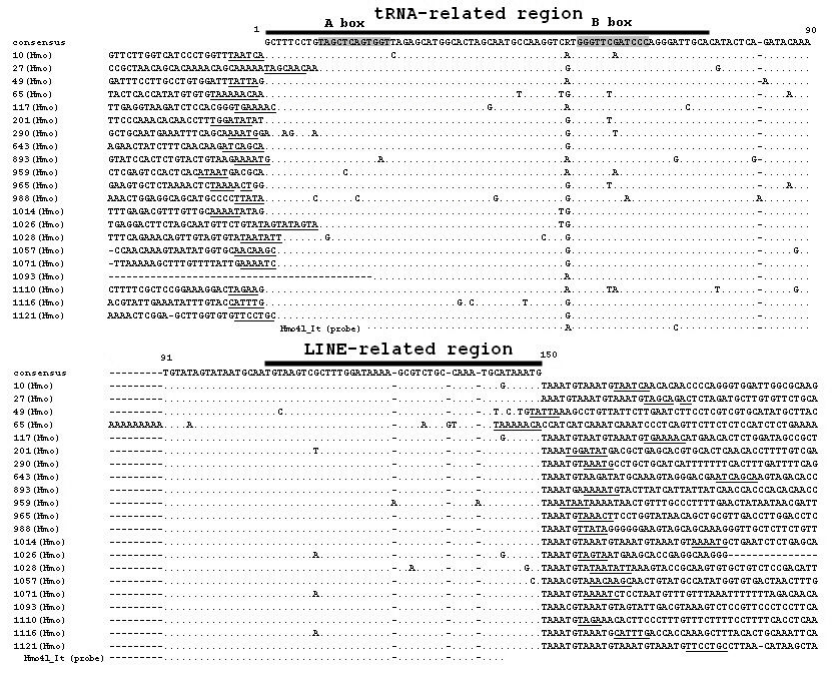
**HAmo SINE from silver carp**. The consensus sequences are shown on top. The tRNA-derived region and LINE-related region of SINE are shown by thick bar. The A box and B box corresponding to consensus sequence are shade. The dots indicate nucleotides identical to the consensus sequence at the top. Underlined nucleotides indicate the target site duplications of each SINE locus. The GenBank accession numbers of HAmo SINE are as follows: FJ171620-FJ171640.

**Figure 5:**
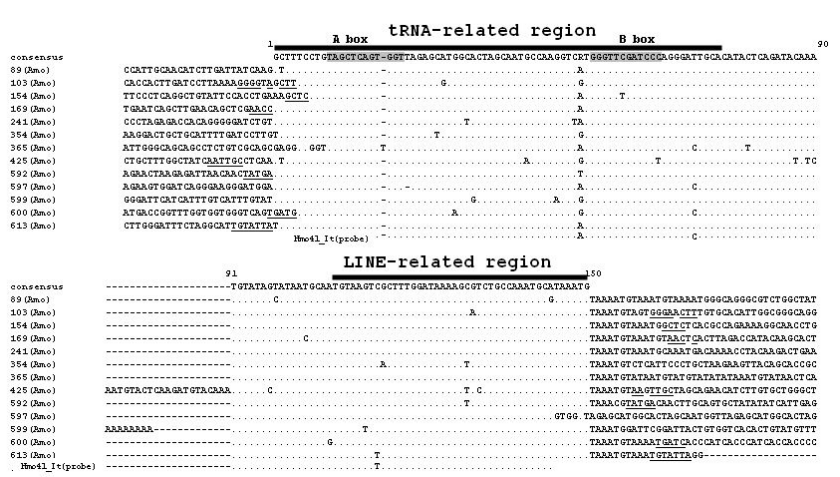
**HAmo SINE from bighead carp**. The caption is identical with figure 4. The GenBank accession numbers of HAmo SINE are as follows: FJ171641-FJ171653.

Using the above non-library enrichment method, we isolated and characterized a new SINE family containing 21 and 13 members from silver carp and bighead carp, respectively. We designated it HAmo SINE family for combining Hmo (*Hypophthalmichthys molitrix*) and Amo (*Aristichthys mobilis*). The consensus sequence of HAmo SINE is 150 bp in length and identical in the two species, which has typical structure of SINEs: a tRNA^Lys^-related promoter region at their 5'-end, a unique central family-specific region and an end with LINE2-derived 3'-terminus preceding the short tandem repeats TAAATG (Figure 4, 5).

We cannot find enough diagnostic nucleotides to divide them into subfamily, and only clone 65 in silver carp and clone 599 in bighead carp shared a common A_9 _insertion in the tRNA-unrelated region. Almost all members in silver carp (except clone1093 with uncompleted 5' sequence) and 7 out of 13 members in bighead carp are flanked by obvious TSD (target site duplications), which are thought to have been produced during retrotransposition. The small sequence divergence among the members of HAmo SINE and SD show that this SINE family seems to be very young and proliferated very recently

### The HAmo SINE 5' End is derived from tRNA

**Figure 6:**
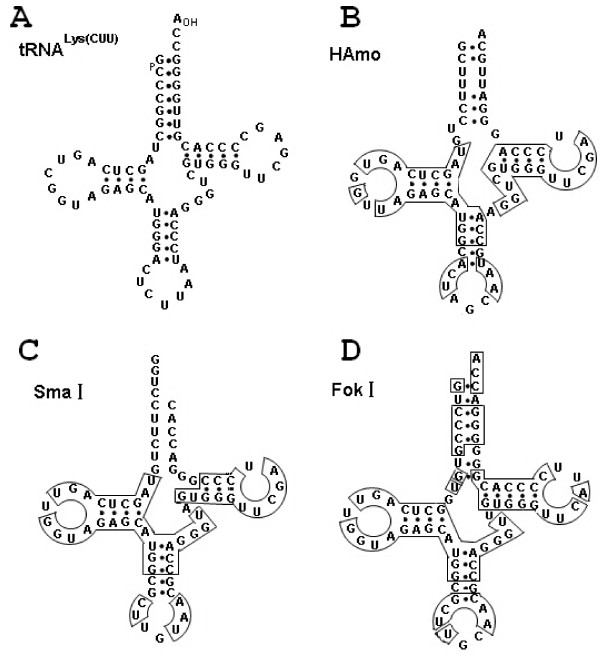
**The tRNA-like structures of tRNA^Lys^(CUU) in rabbit taken from **[39]**(A) and three tRNA^Lys^-related SINE families (B-D)**. The secondary structure of the tRNA-derived region of HAmo SINE family was predicted by tRNAscan-SE program [50]. The tRNA-like structures of SmaI SINE and FokI SINE were reported in elsewhere [40,47]. The identical sequences to tRNA^lys^(CUU) in HAmo SINE, SmaI SINE and FokI SINE are boxed.

BlastN homology search revealed that the tRNA-related region of the HAmo family was most similar to tRNA^Lys ^in Rabbit (83%, not counting the acceptor stem)[39] and showed equal similarity (80%) to tRNA^Lys ^in Rat, chicken, mouse, Bombyx mori, Drosophila melanogaster, respectively. When compared the predicted secondary structure between the tRNA-derived region of HAmo family and Rabbit tRNA^Lys ^(Figure 6), we found a feature that they have no homology in the acceptor stem region, which also happened to tRNA^Lys^-derived SmaI family [40]. However, the significant homology in secondary structures and the numbers of nucleotides in the stem and loop structures suggested that the tRNA^Lys ^species was the most likely candidate for the origin of HAmo SINEs. The obvious conservation of secondary structure in tRNA-related regions including conserved A and B boxes of the split promoter in HAmo SINE manifested their functional importance for transcription by polymerase III.

### Characterization of partner LINE family of HAmo SINE in silver carp and bighead carp

Many isolated clones contain a region only matched to 3' tail of HAmo SINE probe (plasmid Hmo41_It) in the genomes of silver carp and bighead carp when isolating HAmo SINE. After Blast search and alignment of these sequences of clones, we characterize them as 5'HaeIII -fragmented and 5' truncated LINEs, which are homologs of CR1-2_DR in zebrafish. We designated these LINEs as HAmo LINE, since possibly they encode RTase responsible for retrotransposition of HAmo SINE through recognition of the common 3'tail conserved in nucleotide sequences and secondary structure (see next).

**Figure 7:**
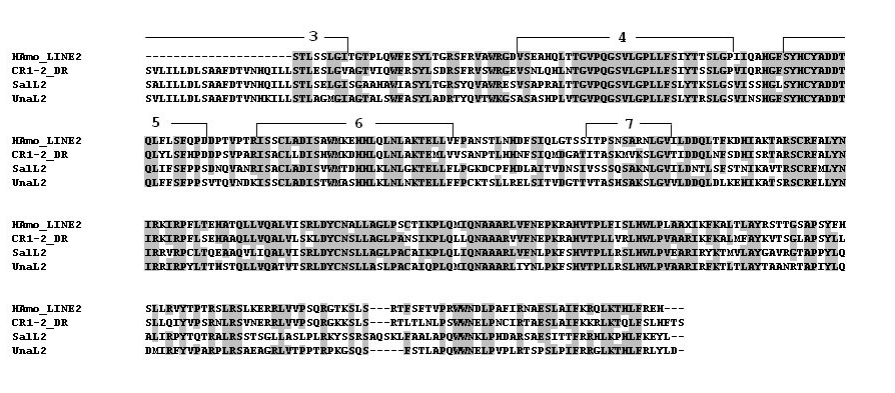
**An alignment of the sequences of the amino acids in the reverse transcriptases encoded by HAmo LINE, CR1-2_DR **[43], **SalL2 **[24], **UnaL2 **[26]. When most of Amino acid residues at a given position are identical, they are shaded. The deduced RT domains shared by these LINE families were denoted.

In order to determine the 5' upstream sequence from a breakpoint at the HaeIII site of these LINEs, we employed the genomic DNA walking method to determine many sequences of clones containing 5'-truncated partial LINE (see Additional file 3). At last, a consensus sequence of 1296 bp was deduced, corresponding to partial ORF that encode RTase and 3' UTR. The predicted partial amino acid sequences encoded by HAmo LINE are 72% identical to that of CR1-2_DR and homologous to that of other LINE2 (Figure 7).

**Figure 8:**
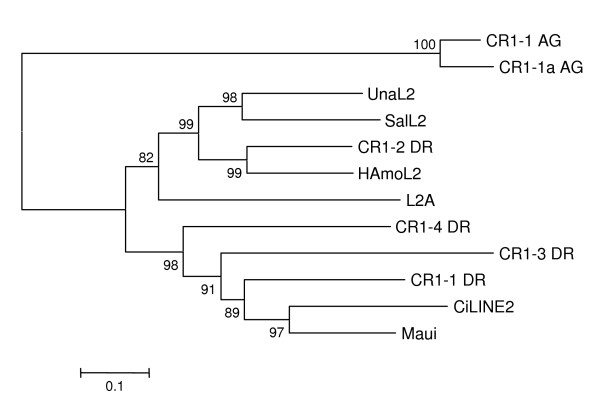
**Phylogenetic relationships between HAmo LINEs and other LINE families belonging to L2 clade**. Analysis was performed using partial RTase of HAmo LINE and full length RTase of other LINEs. The phylogenetic tree was constructed by the neighbor-joining method (with programs in the MEGA4 package [44]). Numbers above the branches indicate the bootstrap values per 1000 replications and provide an indication of the statistical significance of the nodes. Related references of used LINEs are as follows: UnaL2 [26]; L2A (Eutheria *consensus*, RepBase 1999); CiLINE2 [45]; Maui (AAD19348); CR1-2_DR, CR1-1_DR, CR1-3_DR, CR1-4_DR, CR1-1_AG, CR1-1a_AG were obtained from Repbase[43]

Eickbush's group divided all identified LINEs into 11 distinct clades based on an extended sequence alignment of their RT domains [41]. Recently a novel L2 clade is well separated from the CR1 clade which is widely distributed in eukaryotic genomes such as vertebrates, echinoderms and insects [24,42]. When HAmo LINE sequence was added into analysis, the phylogenetic tree shows that HAmo LINE was most close to zebrafish CR1-2_DR and constituted a monophyletic group with zebrafish CR1-2_DR, salmon SalL2 and eel UnaL2 in the L2 clade of LINEs (Figure 8) [43-45]. So HAmo LINE are homolog of other L2 in various distantly related species that diverged over 300 million years ago [24].

### The common tail conserved in primary and secondary structures between HAmo SINE and HAmo LINE

**Figure 9:**
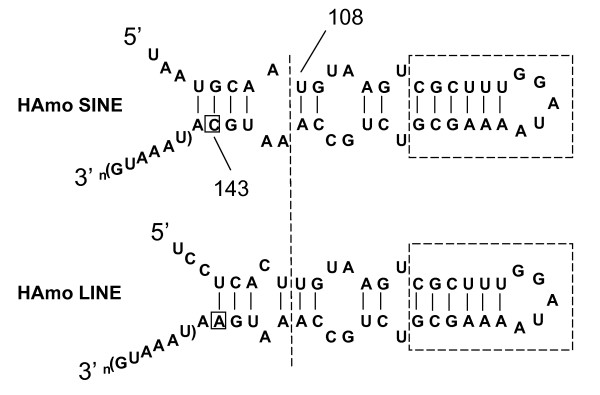
**Putative secondary structures of the tail regions of HAmo SINE and HAmo LINE**. The tail of HAmo SINE shared by HAmo LINE2 and other L2 in sequence starts from 108 bp. The domain has a hairpin region with a GGAUA loop is boxed by a dashed line, which is thought to be a recognition region for the LINE RT in UnaL2 [25,46]. Thin lines show base pairs. In the LINE-related region of HAmo SINE (start from 108 bp), One nucleotide (143 bp) are different from that of HAmo LINE, that are boxed.

**Figure 10:**
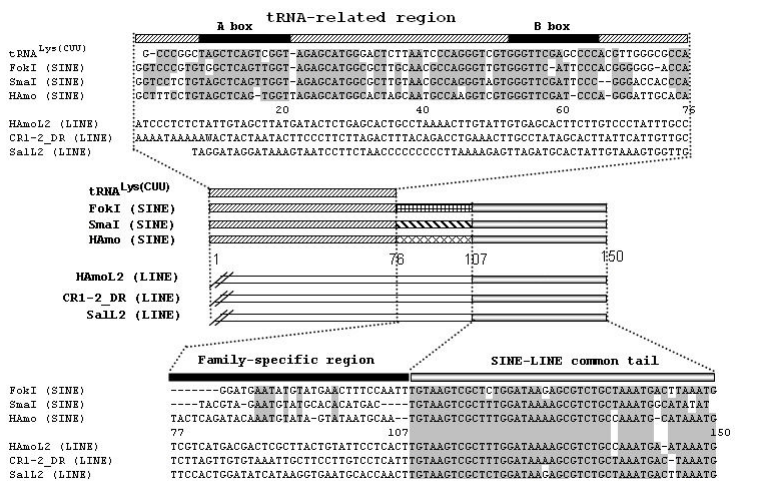
**Comparison of HAmo SINE and two other tRNA^lys^-derived SINE families: SmaI and FokI family **[47-49]. We divided the HAmo SINE into three parts that show different similarity with other two SINEs, respectively. The tRNA-derived region of three SINE families and the tRNA^lys ^(CUU) in rabbit [39] are aligned. The different family-specific regions revealed they were generated independently. The tail regions of these SINEs and their partner LINEs tail are compared. When most of nucleotides at a given position are identical, they are shaded.

HAmo SINE have an approximately 42-bp-long conserved 3'-tail which are almost identical to HAmo LINE and also high similar to other SINE family and their paternal LINE families (see next, Figure 9, Figure 10). There is only one base different in this region between HAmo SINE and HAmo LINE, suggesting that the conserved 3'tail of HAmo SINE, which is important for the process of SINE retrotransposition, is derived from HAmo LINE in the same genomes of silver carp and bighead carp.

The predicted secondary structures for the 3' tail RNA of HAmoL2 and HAmo SINE forms a secondary structure consisting of a stem and a loop (Figure 9). HAmo SINE and HAmo LINE share a hairpin region with a GGAUA loop which is thought to be a recognition domain for the LINE RT in UnaL2 [26,46]. So the clear and significant homolog in the 3'-tail between HAmo SINE and their partner HAmo LINE in primary and secondary structures suggested the HAmo SINE may borrow the enzymatic machinery of HAmo LINE to proliferate in the same genome through the conserved 3' -tail.

Interestingly, short tandem repeats (TAAATG) of variable numbers are observed in the 3'terminus of the tail in both HAmo SINE and HAmo LINE. Most of copies have more than one repetition of the repeat in the tail, which is probably required for the slippage reaction during reverse transcription initiation [25].

### Estimation of copy numbers of HAmo SINE

The pair of internal primer: primer ItF and primer ItR also was used to amplify the HAmo SINE sequences in genomic DNA (sample) and plasmid Hmo41_It (standard) in Real-Time PCR. The results are summarized in Additional file 4. We used a serial of diluted genomic DNA as tested samples to perform PCR reaction, the final estimations of copy numbers using different concentrated genomic DNA template are very close, suggesting that the result of experiment are stable and efficient. At last, average copy numbers of HAmo SINE in haploid genome of silver carp and bighead carp were estimated to 2.22 × 10^5 ^and 1.37 × 10^5^, respectively. Considering the possibility of mismatch between primers with more divergent HAmo SINE sequences, so the results of qRT-PCR were minimal estimates of HAmo SINE copy numbers in the two genomes of silver carp and bighead carp.

## Discussion

### Possible structures of HAmo SINE leading to successful proliferation

The analysis of HAmo SINE shows that it was very young and proliferated recently to estimated about 2 × 10^5 ^and 1.7 × 10^5 ^copy numbers in the haploid genome of silver carp and bighead carp, respectively. In fact, most of the SINE loci isolated in this work are species-specific or even not fixed among fish populations when we detected the presence or absence of SINE insertions using flanking primers (our group, unpublished data). So HAmo SINE are highly efficient and successfully proliferated recently in the genome and it maybe owe to its overall structure and internal structure as described below.

Firstly, HAmo SINE keep the overall secondary structure and conserved A and B box in the tRNA-related region, which ensures the RNA III recognition and transcriptional activity of SINEs. More importantly, the irregularity of the acceptor stem, as same as SmaI family, seems to help to escape recognition by tRNA-processing or RNA-modifying enzymes and therefore prevent the RNA from being cleaved by the 3'-endonuclease.

Secondly, HAmo SINE share the almost identical 3'tail with HAmo LINE2 in primary sequence and secondary structures, which keep them to well utilize the LINE2 enzymatic machinery. Their shared same stem-loop region is thought to function as a recognition site for the UnaL2 protein (UnaL2p) when this region is transcribed in the RNA [46].

Moreover, more than one repetition of the short tandem repeat TAAATG appeared in most copies, which are revealed to be necessary for successful retrotransposition by mutational analyses in the experiments on other LINEs of the L2 clade and the initiation of reverse transcription of UnaL2 RNA in UnaL2 [25,28].

Thirdly, RNA structure of the HAmo SINE is obviously composed of three parts: tRNA-related region, a family-specific region and LINE2-related region (Figure 10), that correspond to there parts of secondary structure of its RNA: the cloverleaf structure (the 5'domain), an unstructured region, the extended stem-loop (the 3'domain). This characteristic domain composition is experimentally probed in salmon SmaI SINE RNA and seems to have guaranteed successful and continuous amplification of SINEs in eukaryotic genomes during evolution. From this view, HAmo SINE may reveal some internal structures of SINE that may lead to its successful retrotransposition and proliferation.

### Three similar but independently derived SINE families

When using HAmo SINE as query to tBlastN search, it shows unexpected high similarity to other two SINE families in salmon: SmaI family (77%) and FokI family (71%), both are young SINE families and have a limited distribution in several specific species belonging to the family Salmonidae [47]. The two SINE families shared a common tail and parasitized SalL2 in salmon genome [24]. After detailed comparison of the consensus sequences of them and their respective partner LINE families (Figure 10)[48,49], we found that HAmo SINE is similar in tRNA-related region (1–76 bp) and LINE-related region (107–150 bp) with SmaI family and FokI family. But the existence of a central region (76–107 bp) which showed no similarity with each other and are specific to each family make us deduce that they are probably independently generated and evolved in respective evolutionary lineage other than horizontal transfer.

As noted in the *Introduction *section, the template switch during TPRT was proposed to explain how SINE acquired the tail from corresponding LINE. In this process, a short cDNA would first be generated by copying the 3'terminal LINE RNA sequence, and then RT landing pad will jump to another RNA parent of the SINE-to-be carrying an internal pol III promoter [34]. So the above-mentioned tRNA^Lys ^derived SINE may be born through template switch between respective LINE and ancestor RNA of SINE-to-be containing tRNA-derived region and family-specific region in respective genome of three fishes. Coincidently, the three young families are all derived from tRNA^Lys ^or structurally related to tRNA^Lys ^(Figure 6)[50]. Moreover, their parental LINE (HAmoL2 and Sal L2) of the above mentioned three SINE families are homologous and share a common tail.

In fact, tRNA^Lys ^is the most common source of SINEs [7,24,51]. The possible reason is that maybe the ancestor tRNA^Lys ^SINE RNA had special selective advantage in the above generation process or been preferentially transcripted and retrotransposed after generation among the population of RNA of SINE -to be.

So this finding suggested that the three similar but distantly related young SINE families were generated independently and created by LINE families within the same lineage of a LINE phylogeny in the genomes of different hosts.

### Some aspects about the new retroposons enrichment strategy

Magnetic Bead-based isolation system has been widely used for the separation of several specific targets like cells, proteins, microsatellites and so on. However, our work is the first report about application of this system for isolation of SINEs and LINEs from fish genomes by developing new special protocol. The results demonstrate that this protocol is technically straightforward and permits the isolation of a large number of SINE and LINE from unknown genome in less time consumption and less cost and effort than is required to execute traditional protocol involving rounds of filter hybridization.

In general, if all steps work, the procedure takes only about a week from tissue to several hundred positive clones. Additionally, the purchase of the reagents needed for building and screening one library by traditional protocol will supply sufficient reagents for ten or more libraries applied by enrichment protocol. Moreover, the protocol can be easily controlled and handled since it requires little specialized equipment platform or technical expertise, May be the PCR and cloning be the most difficult step.

Our method, relying on solution hybridization, could greatly facilitate and speed up the interaction between probe and target DNA and result in better hybridization efficiencies in comparison with fixed solid supports [52,53]. Moreover, this method can be useful in the case of low copy number SINEs and LINEs since at last only a population of sequences enriched for specific retroposons is cloned. Generally the frequency of positive clones can reach 50–90% if conditions were optimized [54]. So it shows great advantage when usually a great number of retroposon insertions need be isolated as temporal landmarks of evolution for estimations of phylogeny.

Most steps in the protocol presented here can be readily modified to suit different experimental backgrounds and knowledge about SINE and LINE and can easily combine with other protocols. Okada's group successfully isolated many SINE families from many organisms by using the in vitro transcript of total genomic DNA as the probes utilizing the properties that SINEs are redundant in the genome and transcribed by RNA polymerase III [55,56]. While Kramerov's group prefered to use AB-PCR product containing a 30–40 bp sequence located between boxes A and B of SINE as a probe [37,57]. All these specific probes including known SINE sequence (this paper) can be biotinylated to join into this enrichment strategy.

But it is noted that there are many principles that should be kept in mind. Firstly, correct restriction enzyme should be selected to generate appropriate size fragments evenly and its recognized sites should not exist in the targeted repeat elements. In our work, although we isolated SINEs and LINEs simultaneously at one isolation reaction, we only obtained the HaeIII-fragmented partial LINEs because of the existed HaeIII site in full-size LINEs. Secondly, the amplification cycles of step *PCR enrichment *and *adapter PCR *(see *Methods*) should be optimized to generate a smear of the PCR products without specific bands. In this case, 15 and 12 of cycles were done in the two steps respectively to keep the complexity of DNA molecules for preventing the generation of a lot of identical clones at last. Moreover, the selectivity and specificity can be adjusted by altering specific probe and the stringency conditions (temperature and salinity of washing buffer). Thirdly, there are several methods for labeling one biotin at one terminus of the DNA fragment such as PCR method with one of the two primers biotinylated (this paper), end-labeling using terminal transferase, ligation reaction with a biotinylated adaptor [58] or direct generated by company service. No matter which method to be used, it is important to label only one biotin molecule at one terminus of the DNA fragment, otherwise magnetic beads will crosslink and clot through DNA bridges, which may result in poor reaction kinetics between beads and target molecules. In addition, isolation of large size of DNA fragments may be limited by beads binding ability and cloning efficiency of large fragments into T-vector. However, our procedures mainly base on PCR and hence could be use to track the progress of the entire process from step to step by gel electrophoresis.

## Conclusion

The young HAmo SINE family and its partner HAmo LINE2 family shared a common 3'tail region in two carp fishes, indicated the retropositional parasitism of HAmo SINEs on HAmo LINEs and strengthened already proposed hypotheses including the formation of the stem loop structure in 3'tail region of some SINEs and LINEs and the mechanism of template switching in generating new SINE family. The finding of repeat sequences in the 3' tail of the HAmo SINEs strengthened the slippage model for initiation of reverse transcription. The obtained results show that the developed new protocol for isolation of SINE and LINE are advantages and technically straightforward. The characterization of new SINE and LINE pair is also beneficial to the future study about the molecular systematics of cyprinid fish.

## Abbreviations

SINE: short interspersed repetitive elements; LINE: long interspersed repetitive elements; qRT-PCR: Quantitative Real Time-PCR; NJ: neighbor joining; TSD: target site duplication; RTase: reverse transcriptase.

## Authors' contributions

SH and CT conceived and designed the experiments, CT and BG performed the experiments and analyzed the data, CT and SH wrote the paper. All authors have read and approved the final manuscript.

## Supplementary Material

Additional file 1 **Scheme of inverse PCR and recovery of the origin sequence.** The figure provided shows and describes the whole procedure of inverse PCR including recovery of origin sequence. Click here for file

Additional file 2 **The efficiency of the novel method for isolation of SINEs and LINEs.** This table provided shows the numbers of different kinds of clones including SINEs and LINEs isolated by the novel method. Click here for file

Additional file 3 **A schematic representation of clones used to determine the consensus sequences of HAmo LINE.** The clones obtained by the retroposons enrichment strategy and by genome walking method are separately located on the right and left. The GenBank accession numbers of HAmo LINE are as follows: FJ171663-FJ171689. Click here for file

Additional file 4 **HAmo_SINE Copy numbers estimated by qRT-PCR.** This table provided shows CT value and estimated HAmo_SINE copy numbers in serial dilutions of standard plasmid and sample DNA in detail. Click here for file
