# Supplementary material for: Bead-probe complex capture a couple of SINE and LINE family from genomes of two closely related species of East Asian cyprinid directly using magnetic separation
Source: BMC Genomics. 2009 Feb 19;10:83. doi: 10.1186/1471-2164-10-83 (PMC2653535; doi:10.1186/1471-2164-10-83)
Supplement: Additional file 1 — Scheme of inverse PCR and recovery of the origin sequence. The figure provided shows and describes the whole procedure of inverse PCR including recovery of origin sequence. [file 1471-2164-10-83-S1.ppt]

## Slide 1
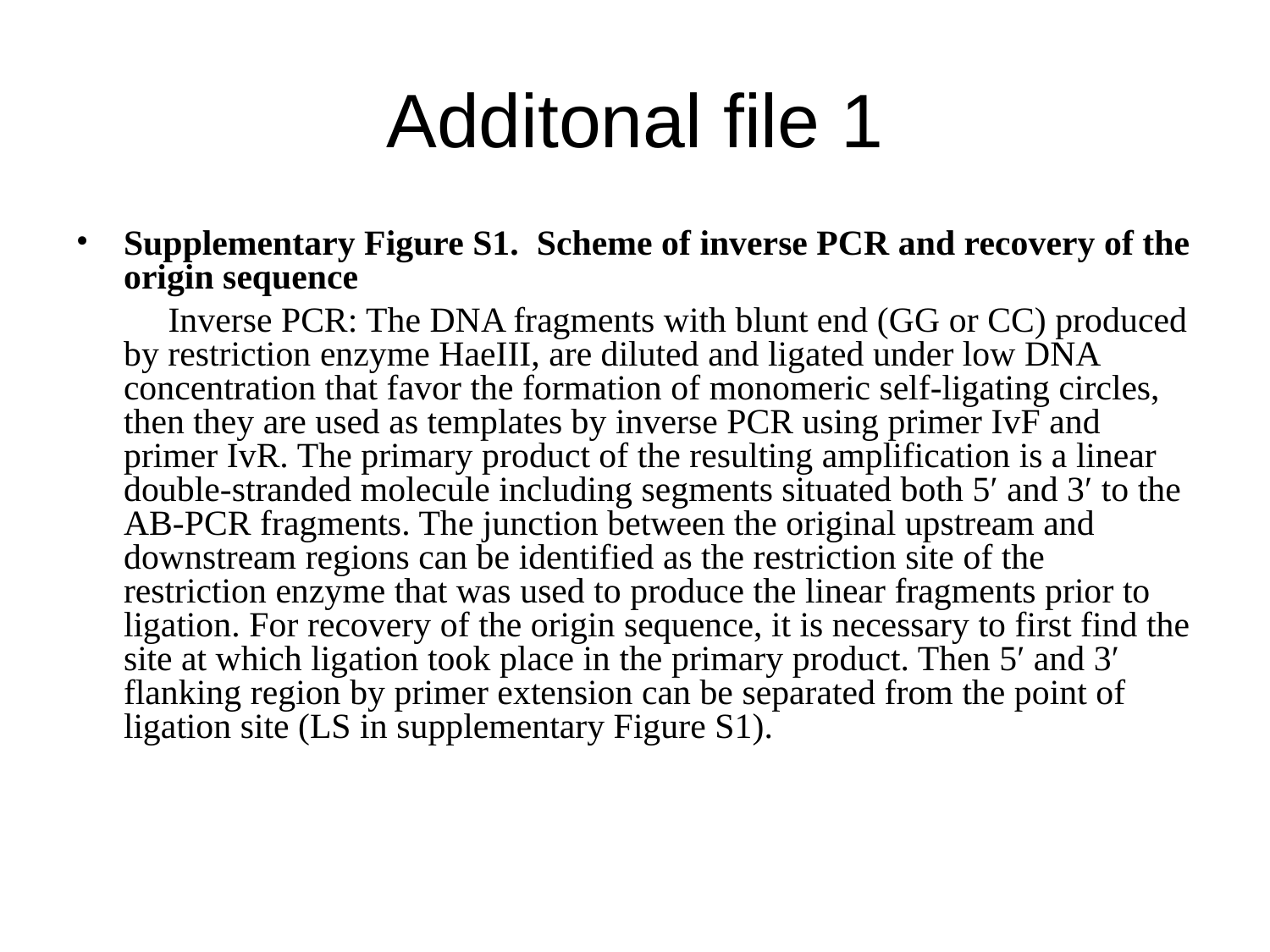

# Additonal file 1
Supplementary Figure S1. Scheme of inverse PCR and recovery of the origin sequence
 Inverse PCR: The DNA fragments with blunt end (GG or CC) produced by restriction enzyme HaeIII, are diluted and ligated under low DNA concentration that favor the formation of monomeric self-ligating circles, then they are used as templates by inverse PCR using primer IvF and primer IvR. The primary product of the resulting amplification is a linear double-stranded molecule including segments situated both 5′ and 3′ to the AB-PCR fragments. The junction between the original upstream and downstream regions can be identified as the restriction site of the restriction enzyme that was used to produce the linear fragments prior to ligation. For recovery of the origin sequence, it is necessary to first find the site at which ligation took place in the primary product. Then 5′ and 3′ flanking region by primer extension can be separated from the point of ligation site (LS in supplementary Figure S1).

## Slide 2
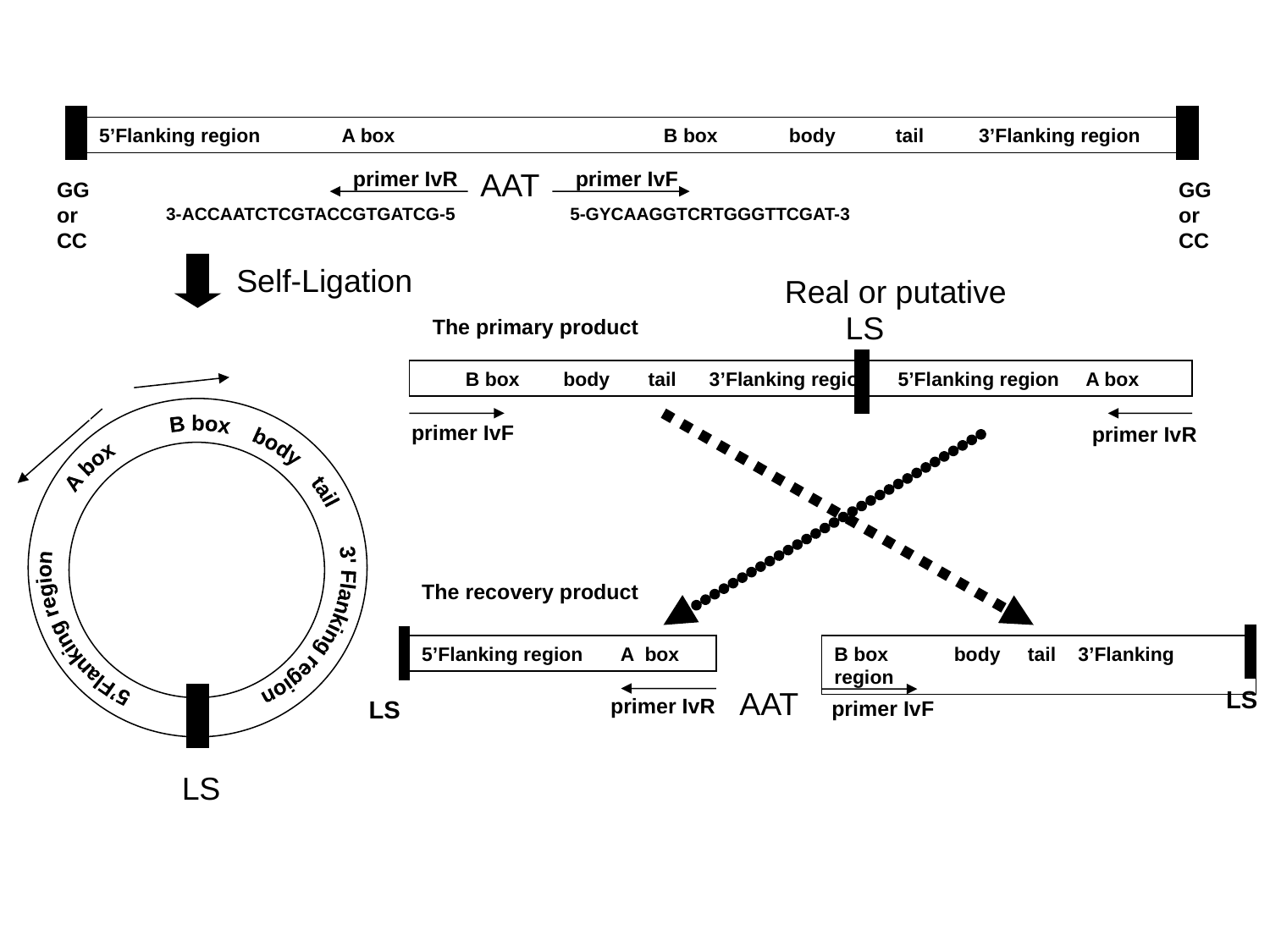

5’Flanking region A box B box body tail 3’Flanking region
primer IvR
AAT
primer IvF
GG or CC
GG or CC
3-ACCAATCTCGTACCGTGATCG-5
5-GYCAAGGTCRTGGGTTCGAT-3
LS
Self-Ligation
Real or putative
The primary product
primer IF
 B box body tail 3’Flanking region 5’Flanking region A box
primer IR
primer IvF
primer IvR
5’Flanking region A box B box body tail 3' Flanking region
The recovery product
5’Flanking region A box
B box body tail 3’Flanking region
AAT
LS
primer IvR
LS
primer IvF
LS
