# Supplementary material for: Bead-probe complex capture a couple of SINE and LINE family from genomes of two closely related species of East Asian cyprinid directly using magnetic separation
Source: BMC Genomics. 2009 Feb 19;10:83. doi: 10.1186/1471-2164-10-83 (PMC2653535; doi:10.1186/1471-2164-10-83)
Supplement: Additional file 2 — The efficiency of the novel method for isolation of SINEs and LINEs. This table provided shows the numbers of different kinds of clones including SINEs and LINEs isolated by the novel method. [file 1471-2164-10-83-S2.doc]

**The efficiency of the novel method for isolation of SINEs and LINEs**

| Species | Total sequenced  clones | Hybridized  clones | Identical  clones | unique positive  clones | Efficiency  (ratio of unique positive clones ) | SINE | LINE | undetermined |
| --- | --- | --- | --- | --- | --- | --- | --- | --- |
| Silver carp | 89 | 59 | 8 | 51 | 57.3% | 27 | 21 | 3 |
| Bighead carp | 49 | 32 | 3 | 29 | 59.2% | 22 | 7 | 0 |
