# Supplementary material for: Bead-probe complex capture a couple of SINE and LINE family from genomes of two closely related species of East Asian cyprinid directly using magnetic separation
Source: BMC Genomics. 2009 Feb 19;10:83. doi: 10.1186/1471-2164-10-83 (PMC2653535; doi:10.1186/1471-2164-10-83)
Supplement: Additional file 3 — A schematic representation of clones used to determine the consensus sequences of HAmo LINE. The clones obtained by the retroposons enrichment strategy and by genome walking method are separately located on the right and left. The GenBank accession numbers of HAmo LINE are as follows: FJ171663-FJ171689. [file 1471-2164-10-83-S3.ppt]

## Slide 1
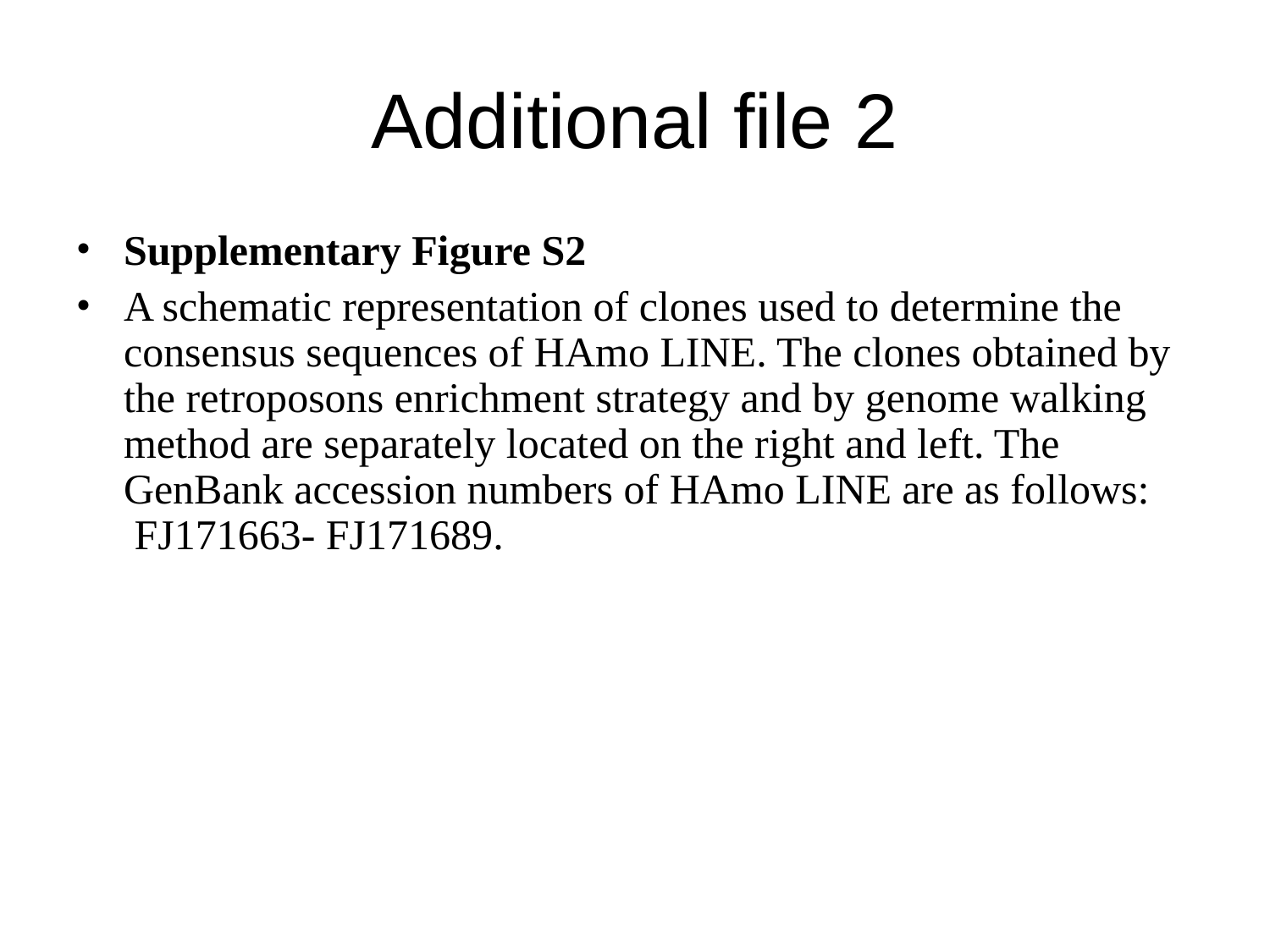

# Additional file 2
Supplementary Figure S2
A schematic representation of clones used to determine the consensus sequences of HAmo LINE. The clones obtained by the retroposons enrichment strategy and by genome walking method are separately located on the right and left. The GenBank accession numbers of HAmo LINE are as follows:  FJ171663- FJ171689.

## Slide 2
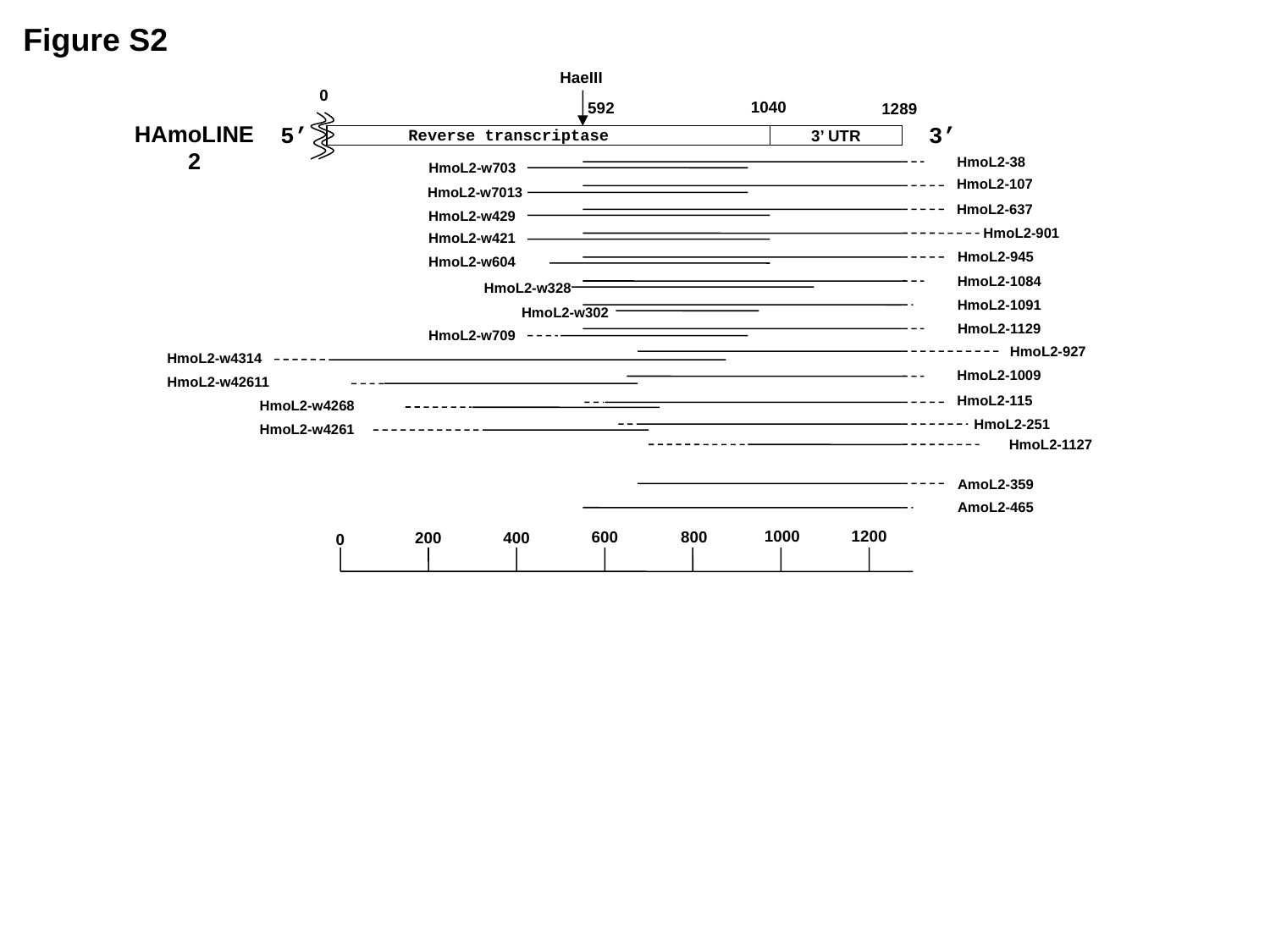

Figure S2
HaeIII
0
1040
592
1289
HAmoLINE2
5’
3’
Reverse transcriptase
3’ UTR
HmoL2-38
HmoL2-w703
HmoL2-107
HmoL2-w7013
HmoL2-637
HmoL2-w429
HmoL2-901
HmoL2-w421
HmoL2-945
HmoL2-w604
HmoL2-1084
HmoL2-w328
HmoL2-1091
HmoL2-w302
HmoL2-1129
HmoL2-w709
HmoL2-927
HmoL2-w4314
HmoL2-1009
HmoL2-w42611
HmoL2-115
HmoL2-w4268
HmoL2-251
HmoL2-w4261
HmoL2-1127
AmoL2-359
AmoL2-465
1000
1200
600
800
200
400
0
