# Supplementary material for: Bead-probe complex capture a couple of SINE and LINE family from genomes of two closely related species of East Asian cyprinid directly using magnetic separation
Source: BMC Genomics. 2009 Feb 19;10:83. doi: 10.1186/1471-2164-10-83 (PMC2653535; doi:10.1186/1471-2164-10-83)
Supplement: Additional file 4 — HAmo_SINE Copy numbers estimated by qRT-PCR. This table provided shows CT value and estimated HAmo_SINE copy numbers in serial dilutions of standard plasmid and sample DNA in detail. [file 1471-2164-10-83-S4.doc]

**HAmo_SINE Copy numbers estimated by qRT-PCR**

|  |  | ×1 (Con) |  | Serial dilutions of standard plasmid and sample DNA | | | | |  |
| --- | --- | --- | --- | --- | --- | --- | --- | --- | --- |
|  |  | /DNA Size |  | ×1 | ×10^-1^ | ×10^-2^ | ×10^-3^ | ×10^-4^ |  |
| Plasmid  Hmo41_It | standard | 2×10**^-4^**ug/ul  2.82X10^3^bp | CT | 10.35 | 13.11 | 15.91 | 19.86 | 23.42 | SC: Y＝-0.299×CT+6.92  R^2^=0.997; E=0.99 |
|  |  |  |  |  |  |  |  |  |  |
| Silver carp | sample | 1.52ug/ul | CT | 11.15 | 14.22 | 17.67 | 20.98 | 23.79 | Average copy no. per haploid genome |
| Genomic DNA |  | ≈1X10^9^bp | PCNH | 1.80×10^5^ | 2.18×10^5^ | 2.03×10^5^ | 2.08×10^5^ | 3.0×10^5^ | 2.22×10^5^ |
|  |  |  |  |  |  |  |  |  |  |
| Bighead carp | sample | 2.73ug/ul | CT | 11.1 | 14.15 | 17.22 | 20.70 | 23.85 | Average copy no. per haploid genome |
| Genomic DNA |  | ≈1X10^9^bp | PCNH | 1.04×10^5^ | 1.28×10^5^ | 1.54×10^5^ | 1.40×10^5^ | 1.61×10^5^ | 1.37×10^5^ |

Con: concentration; CT: (cycle threshold) is defined as the number of cycles required for the fluorescent signal to cross the threshold; PCNH: Predicted copy no. in haploid genome; SC: standard curve; The R^2^ value is the coefficient that is used to assess the fit of the standard curve to the data points plotted. The R2 value was >0.99 which is the required value for reliable quantitation. The efficiency of the PCR reaction (E) is calculated using the formula E= (10^(−1 / slope)^−1), where the slope is calculated from a standard curve plot of Ct values against the logarithm of template amount. A value close to 1 indicates high PCR efficiency. PCNH was calculated using the equation: PCNH= genomic DNA size (bp) / Plasmid DNA size (bp) × 2×10**^-4^** (μg/μl) / 10^0^ × 10^Y^ / Genomic DNA Con (μg/μl)
